# Glucose concentrations of less than 3.0 mmol/l (54 mg/dl) should be reported in clinical trials: a joint position statement of the American Diabetes Association and the Europian Association for the Study of Diabetes

**DOI:** 10.1007/s00125-016-4146-6

**Published:** 2016-11-21

**Authors:** The International Hypoglycaemia Study Group

**Affiliations:** c/o Simon R. Heller, Department of Oncology and Metabolism, University of Sheffield, Medical School, Beech Hill Road, S10 2RX Sheffield, UK

**Keywords:** Glucose Concentration, American Diabetes Association, Severe Hypoglycaemia, Plasma Glucose Concentration, Continuous Glucose Monitoring

The International Hypoglycaemia Study Group recommends that the frequency of detection of a glucose concentration <3.0 mmol/l (<54 mg/dl), which it considers to be clinically significant biochemical hypoglycaemia, be included in reports of clinical trials of glucose-lowering drugs evaluated for the treatment of diabetes mellitus.

The glycaemic thresholds for symptoms of hypoglycaemia and for glucose counterregulatory (including sympathoadrenal) responses to hypoglycaemia, as plasma glucose concentrations fall, are not fixed in patients with insulin-, sulfonylurea- or meglitinide- (glinide)-treated diabetes. They are at higher glucose concentrations in those with poor glycaemic control and at lower glucose concentrations in those with tight glycaemic control [1–5]. The shifts in glycaemic threshold to lower glucose concentrations are largely the result of more frequent episodes of iatrogenic hypoglycaemia during intensive glycaemic therapy. Glycaemic thresholds for responses to hypoglycaemia vary, not only among individuals with diabetes but also in the same individual with diabetes as a function of their HbA_1c_ levels and hypoglycaemic experience; it is therefore not appropriate to cite a specific glucose concentration that defines hypoglycaemia in diabetes. As a consequence, the American Diabetes Association has defined hypoglycaemia in diabetes non-numerically as ‘all episodes of an abnormally low plasma glucose concentration that expose the individual to potential harm’ [6, 7].

Nonetheless, the International Hypoglycaemia Study Group believes that it is important to identify and record a level of hypoglycaemia that needs to be avoided because of its immediate and long-term danger to the individual. A single glucose level should be agreed to that has serious clinical and health-economic consequences. This would enable the diabetes and regulatory communities to compare the effectiveness of interventions in reducing hypoglycaemia, be they pharmacological, technological or educational. It would also permit the use of meta-analysis as a statistical tool to increase power when comparing interventions.

In its discussion, the International Hypoglycaemia Study Group considered glucose concentration levels of <3.0 mmol/l (<54 mg/dl) and <2.8 mmol/l (<50 mg/dl) detected by self-monitoring of plasma glucose, continuous glucose monitoring (for at least 20 min) or a laboratory measurement of plasma glucose. Both of these levels are distinctly low glucose concentrations that do not occur under physiological conditions in non-diabetic individuals [8]. Thus, they are unequivocally hypoglycaemic values. They approximate the upper and lower limits, respectively, of the non-diabetic glycaemic threshold for symptoms of insulin-induced hypoglycaemia [8–10]. The generic non-diabetic glycaemic threshold for impairment of cognitive function is <2.8 mmol/l [8–10], but higher glucose levels have been reported for some tests [11–14]. Glucose concentrations of both <3.0 mmol/l and <2.8 mmol/l cause defective glucose counterregulation and impaired awareness of hypoglycaemia, the core components of hypoglycaemia-associated autonomic failure in diabetes [5]. Avoiding these glucose levels could reverse impaired awareness of hypoglycaemia [15–18] and some aspects of defective glucose counterregulation [15–17] in many affected patients. In type 1 diabetes, failure to recognise one’s own hypoglycaemia at a glucose concentration <3.0 mmol/l increased the risk of severe hypoglycaemia (defined as needing the help of another person for recovery) fourfold [17]. In type 2 diabetes, both glucose concentrations were associated with cardiac arrhythmias [19, 20]. Finally, a glucose concentration <2.8 mmol/l was associated with mortality in patients with type 2 diabetes in the Action to Control Cardiovascular Risk in Diabetes (ACCORD) trial (NCT00000620) [21], and possibly in the Outcomes Reduction with an Initial Glargine Intervention (ORIGIN) trial (NCT00069784) [22] and among patients treated in intensive care units in the Normoglycemia in Intensive Care Evaluation—Survival Using Glucose Algorithm Regulation (NICE-SUGAR) trial (NCT00220987) [23]. A glucose concentration <3.0 mmol/l was associated with mortality in the NICE-SUGAR trial [23] and, possibly, in the ORIGIN trial [22].

Ultimately, the International Hypoglycaemia Study Group members agreed that a glucose concentration <3.0 mmol/l (<54 mg/dl) is sufficiently low to indicate serious, clinically important hypoglycaemia. Possible terms used to describe this condition include ‘serious’, ‘clinically important’, ‘major’ or ‘clinically significant’. The group decided not to describe ‘severe hypoglycaemia’ in terms of glucose concentration since there is currently widespread agreement that severe hypoglycaemia, as defined by the American Diabetes Association [6, 7], denotes severe cognitive impairment requiring external assistance for recovery. The group also proposed that the frequency of detection of the glucose alert value of 3.9 mmol/l (70 mg/dl) or less [24] need not be reported routinely in clinical trials.

In conclusion we propose that the following glucose levels be adopted by the diabetes community to address the issue of hypoglycaemic risk (text box).

## Appendix

### Members of the International Hypoglycaemia Study Group

Stephanie A. Amiel, RD Lawrence Professor of Diabetic Medicine, Division of Diabetes and Nutritional Sciences, King’s College London, London, UK

Pablo Aschner, Associate Professor of Endocrinology, Javeriana University School of Medicine, Director of Research, San Ignacio University Hospital and Scientific Director of the Colombian Diabetes Association, Bogotá, Colombia

Belinda Childs RN, Executive Director, Clinical Nurse Specialist, Great Plains Diabetes, Wichita, KS, USA

Philip E. Cryer, Professor of Medicine Emeritus, Washington University in St Louis, St Louis, MO, USA

Bastiaan E. de Galan, Department of Internal Medicine, Radboud University Nijmegen Medical Centre, Nijmegen, the Netherlands

Simon R. Heller, Professor of Clinical Diabetes, University of Sheffield, and Director of Research and Development and Honorary Consultant Physician, Sheffield Teaching Hospitals NHS Foundation Trust, Sheffield, UK

Brian M. Frier, Honorary Professor of Diabetes, The Queen’s Medical Research Institute, University of Edinburgh, Edinburgh, Scotland, UK

Linda Gonder-Frederick, Associate Professor, Department of Psychiatry and Neurobehavioral Sciences, and Clinical Director, Behavioral Medicine Center, University of Virginia Health System, Charlottesville, VA, USA

Timothy Jones, Clinical Professor, School of Paediatrics and Child Health, Telethon Institute for Child Health Research, University of Western Australia, and Head, Department of Endocrinology and Diabetes, Princess Margaret Hospital for Children, Perth, WA, Australia

Kamlesh Khunti, Professor of Primary Care Diabetes and Vascular Medicine, University of Leicester, Leicester, UK

Lawrence A. Leiter, Division of Endocrinology and Metabolism, St. Michael’s Hospital and Professor of Medicine and Nutritional Sciences, University of Toronto, Toronto, ON, Canada

Rory J. McCrimmon, Professor of Experimental Diabetes and Metabolism, Division of Molecular & Clinical Medicine, School of Medicine, University of Dundee, Dundee, Scotland, UK

Yingying Luo, Associate Professor, Endocrinology and Metabolism Department, Peking University People’s Hospital, Beijing, China

Elizabeth R. Seaquist, Pennock Family Chair in Diabetes Research, Professor of Medicine and Director, Division of Endocrinology and Diabetes, Department of Medicine, University of Minnesota, Minneapolis, MN, USA

Robert Vigersky, Medical Director, Medtronic Diabetes, Washington DC, USA and Professor of Medicine, Uniformed Services University of the Health Sciences, Bethesda, MD, USA

Sophia Zoungas, Professor of Diabetes, Vascular Health and Ageing, School of Public Health and Preventive Medicine, Monash University, Melbourne, VIC, Australia
